# Determination of weight loss methods and effects among wrestlers before an official championship

**DOI:** 10.3389/fnut.2024.1505759

**Published:** 2024-12-06

**Authors:** Ramazan Seker, Ozkan Isik, Erdil Durukan, Meric Eraslan, Laurentiu-Gabriel Talaghir, Viorel Dorgan

**Affiliations:** ^1^Faculty of Sport Sciences, Hatay Mustafa Kemal University, Hatay, Türkiye; ^2^Faculty of Sports Sciences, Balıkesir University, Balikesir, Türkiye; ^3^Faculty of Sports Sciences, Akdeniz University, Antalya, Türkiye; ^4^Faculty of Physical Education and Sport, Dunarea de Jos University of Galati, Galati, Romania

**Keywords:** dehydration, ergogenic aids, food restriction, rapid weight loss, wrestling

## Abstract

**Introduction:**

It is known that combat athletes frequently lose weight before a competition. This study aimed to determine the weight loss methods before an official championship and the effects of these weight loss methods on the performance of wrestlers.

**Method:**

The sample of the study consisted of 350 competitive wrestlers. The “Athlete Weight Loss Methodology and Effects Scale” and personal information form were used as data collection tools in the study. In the data analysis, independent samples T-test, One-way ANOVA, and Pearson correlation analysis were used in addition to descriptive statistics.

**Results:**

Wrestlers generally reported dieting by restricting fatty (89.1%) and carbohydrate (83.4%) foods. It was observed that they preferred jogging with a raincoat (89.1%) and/or using the sauna (79.7%) as a weight loss method. Wrestlers also reported that they performed weight loss, although at a low level, by using ergogenic aids such as laxatives (31.7%) and/or diuretic pills (28.0%). They were observed to experience physiological effects such as muscle cramps (72.9%), injury (71.1%), and/or difficulty breathing (60.9%), as well as psychological effects such as feeling extremely tired (81.7%), stress (79.7%), decreased performance (78.6%) and/or extremely irritability (71.4%). It was determined that there was no difference between the weight loss methods and effects of the wrestlers according to their gender and wrestling style (*p* > 0.05). On the other hand, it was determined that the diet level of U20 wrestlers was higher than U17 (*p* < 0.05) and Senior wrestlers and that U17 and U20 wrestlers were exposed to higher levels of physiological and psychological effects than Senior wrestlers (*p* < 0.05).

**Conclusion:**

It was determined that wrestlers preferred methods such as restricting food and fluids, using a sauna, and jogging with a raincoat to lose weight. It is necessary to prevent young wrestlers from losing weight before the competition. Thus, it is possible to prevent wrestlers in their development period (U17 and U20) from being exposed to physiological and psychological effects caused by losing weight.

## 1 Introduction

Nearly 22% of the total medals at the 2024 Paris Olympic Games were awarded in combat sports. A total of 232 medals were awarded in five different combat sports (boxing, fencing, judo, taekwondo, and wrestling) at the 2024 Paris Olympic Games, and wrestling had the most medals in combat sports with 72 medals (1). Although wrestling has recently (before the 2020 Tokyo Olympic Games) faced the threat of being dropped from the Olympic Games, wrestling as one of the oldest combat sports has maintained a place in the modern Olympic Games since the Ancient Greek Olympic Games of 708 B.C.E (2). In the modern Olympics, there are three different wrestling styles: Greco-Roman (GR), Free-Style (FS), and women’s wrestling (WW) (3).

To prevent unfair competition, wrestlers are matched according to their body weight and gender (4). Therefore, to gain a competitive advantage against weaker and smaller opponents than themselves, many athletes lose weight rapidly before the competition (5). Although the negative effects of Rapid Weight Loss (RWL) on health status (athletic performance, body composition, competitive success, and injury, etc.) are well reported (6–9), previous studies have reported that 60–90% of high school, collegiate, and/or international level wrestlers experience RWL (10–13).

Various RWL methods such as food and fluid restriction, using a sauna, jogging in a raincoat, and using laxatives, diet pills and/or diuretics are widely used by wrestlers. Physical (body weight, body mass index, and body composition, etc.) and physiological (basal metabolic rate, total body water, free-fat mass, and fat mass, etc.) changes occur in the organism due to food and fluid restriction (14–16). Using a sauna and jogging in a raincoat accelerates fluid excretion from the body through sweat, and the decrease in body water in both intracellular and extracellular spaces increases physiological stress in the human body (17). Moreover, Isik et al. (18) reported that wrestlers may be exposed to hyperosmolar pressure and may exhibit hypernatremic responses (thirst, increased urine concentration, muscle cramps, dry skin and mouth, confusion, seizures or coma, etc.) due to increased sodium levels depending to decreased fluid levels in the body. Although the use of diuretics is not a preferred method due to being on the WADA list, it is a method preferred by non-Olympic wrestlers for RWL from time to time in local or championships without doping control. RWL with all these methods can affect wrestlers physiologically and expose them to many negative psychological effects. RWL has been reported to cause psychological effects in athletes such as decreased short-term memory, vigor, concentration, and self-esteem, as well as increased confusion, anger, fatigue, depression, and isolation, all of which can hamper competitive performance. For example, decreased short-term memory can impact the ability of an athlete to follow his/her coach’s instructions before a match. Likewise, the lack of concentration and focus can affect the athlete’s ability to deal with distractions during high-level competitions, resulting in poor performance. A low self-esteem may result in difficulty in considering the possibility of winning a match, especially against high-level opponents. Confusion can negatively affect the capacity to make decisions during a competition and anger may result in a lack of control and, despite the importance of aggressiveness in combat sports, excessive anger may increase the possibility of illegal actions (19).

In order to eliminate all these physiological and psychological effects and to prioritize human health, United World Wrestling (UWW) has tried to take various precautions such as reducing the ~16-h period between the weigh-in time and the competition time to 2 h and finishing all competitions (except repackages and medal matches) in one day, but UWW has not been able to prevent it completely. Therefore, despite all these protective rule changes made by UWW, it is important to determine the weight loss methods of wrestlers and the effects of these methods on wrestlers. In this context, this study aimed to determine the weight loss methods of female and male wrestlers competing in different age categories and wrestling styles before an official competition and to reveal the possible physiological and psychological effects of these weight loss methods.

## 2 Methods

### 2.1 Research design and participants

This study was a cross-sectional study, and a purposeful sampling method was used in the selection of participants. Three hundred and eighty-eight wrestlers were initially included in the study. Thirty-eight of the wrestlers were excluded from the study because they did not lose weight. Therefore, the study sample was included in three hundred and fifty wrestlers.

### 2.2 Data collection tools

In the study, data was obtained from wrestlers voluntarily using the online survey method via Google Forms. In addition to the personal information form, the athlete weight loss methods and effects scale were used as measurement tools.

#### 2.2.1 Personal information form

The personal information form included questionaries asking about the wrestlers’ gender, wrestling style, age category, how many days before the competition they lost weight, and the amount of weight loss.

#### 2.2.2 Athlete weight loss methodology and effects scale

To determine the weight loss methods of wrestlers and the possible effects of these methods, the “Athlete weight loss methodology and effects scale” developed by Yarar et al. (20) and a personal information form were used. The Athlete weight loss methodology and effects scale was a five-point Likert-type (1 = Never, 2 = Rarely, 3 = Sometimes, 4 = Frequently, 5 = Always) scale consisting of nineteen questions divided into five sub-dimensions as diet (3 items), dehydration (3 items), ergogenic aids (3 items), physiological effect (5 items), and psychological effect (5 items). The reliability coefficient (Cronbach’s alpha) for the original Athlete weight loss methodology and effects scale was 0.74. In this study, Cronbach’s alpha coefficient for Athlete weight loss methodology and effects scale was determined as 0.81. This result shows that the Athlete weight loss methodology and effects scale was reliable for our study sample.

### 2.3 Ethical approval

This study was ethically approved by the Balıkesir University Health Sciences Non-invasive Research Ethics Committee with the decision numbered 2024/137. The parents of the U18 wrestlers who participated in the study were not in the competition area. For this reason, legal permission was obtained from the coaches of the U18 wrestlers as the legal representatives for data collection. In addition, informed consent was approved from all participants before answering the questions.

### 2.4 Statistical analysis

In addition to descriptive statistics (i.e., percent and frequency), the Kolmogorov–Smirnov test was used for the normality test. In the analysis of normally distributed data, the independent samples t-test was used for comparison according to gender. One-way ANOVA was used to compare wrestling style and age categories. The LSD post-hoc test was applied to determine the source of the difference between groups of age categories. A Pearson correlation analysis was used to determine the relationship between sub-dimensions of weight loss methods and effects scale. Significance was set at *p* < 0.05 and *p* < 0.01, respectively.

## 3 Results

Wrestlers who followed a diet to lose weight reported that they reduced their total food consumption by 79.7%, and specifically reduced their dietary fat and carbohydrate consumption by 89.1 and 83.4%, respectively. When the dehydration methods used by wrestlers to lose weight were examined, it was determined that 89.1% of them jogged with a raincoat, 79.7% used saunas and only 26% tried to lose weight by spitting. When the ergogenic aids consumed by wrestlers to lose weight were examined, it was determined that 31.7% used laxatives, 27.1% used diet pills, and 28.0% tried to lose weight by using diuretic pills (Table 1).

**Table 1:** Examination of weight loss methods in an official competition among wrestlers.

Items of diet sub-dimensions	Likert	f	%	No/Yes
Item 1: I reduce food consumption	Never	71	20.3	20.3
	Rarely	114	32.6	79.7
	Sometimes	96	27.4	
	Often	52	14.9	
	Always	17	4.9	
Item 2: I reduce the consumption of carbohydrate foods	Never	38	10.9	10.9
	Rarely	139	39.7	89.1
	Sometimes	96	27.4	
	Often	53	15.1	
	Always	24	6.9	
Item 3: I reduce the consumption of fatty food	Never	30	8.6	8.6
	Rarely	115	32.9	83.4
	Sometimes	95	27.1	
	Often	79	22.6	
	Always	31	8.9	
Items of dehydration sub-dimensions	Likert	f	%	No/Yes
Item 4: I jog with a raincoat	Never	38	10.9	10.9
	Rarely	93	26.6	89.1
	Sometimes	107	30.6	
	Often	68	19.4	
	Always	44	12.6	
Item 5: I go to the sauna	Never	71	20.3	20.3
	Rarely	108	30.9	79.7
	Sometimes	97	27.7	
	Often	58	16.6	
	Always	16	4.6	
Item 6: I lose weight by spitting	Never	259	74.0	74.0
	Rarely	45	12.9	26.0
	Sometimes	28	8.0	
	Often	13	3.7	
	Always	5	1.4	
Items of ergogenic aids sub-dimensions	Likert	f	%	No/Yes
Item 7: I use laxatives (diarrheals)	Never	239	68.3	68.3
	Rarely	62	17.7	31.7
	Sometimes	35	10.0	
	Often	11	3.1	
	Always	3	0.9	
Item 8: I use diet pills	Never	255	72.9	72.9
	Rarely	54	15.4	27.1
	Sometimes	27	7.7	
	Often	11	3.1	
	Always	3	0.9	
Item 9: I use diuretic pills	Never	252	72.0	72.0
	Rarely	53	15.1	28.0
	Sometimes	31	8.9	
	Often	13	3.7	
	Always	1	0.3	

When the physiological effects of weight loss were examined in wrestlers, they reported that they were exposed to muscle cramps, injuries, difficulty breathing, increased body temperature, and heart palpitations with the prevalence of 72.9, 71.1, 60.9, 57.3, and 48.0%, respectively. When the psychological effects of weight loss were examined in wrestlers, they reported that they were exposed to extremely tired, stressed, decreased performance, extremely irritable, and decreased desire to do sports by 81.7, 79.7, 78.6, 71.4, and 66.3%, respectively (Table 2).

**Table 2:** Examination of weight loss effects in an official competition among wrestlers.

Items of physiological effect sub-dimensions	Likert	f	%	No/Yes
Item 10. I experience muscle cramps	Never	95	27.1	27.1
	Rarely	107	30.6	72.9
	Sometimes	92	26.3	
	Often	43	12.3	
	Always	13	3.7	
Item 11. My body temperature increases (I get a fever)	Never	150	42.9	42.9
	Rarely	105	30.0	57.3
	Sometimes	60	17.1	
	Often	25	7.1	
	Always	10	2.9	
Item 12. I experience heart palpitations	Never	182	52.0	52.0
	Rarely	97	27.7	48.0
	Sometimes	51	14.6	
	Often	13	3.7	
	Always	7	2.0	
Item 13. I experience difficulty breathing	Never	137	39.1	39.1
	Rarely	129	36.9	60.9
	Sometimes	57	16.3	
	Often	20	5.7	
	Always	7	2.0	
Item 14. I experience injury	Never	101	28.9	28.9
	Rarely	121	34.6	71.1
	Sometimes	84	24.0	
	Often	30	8.6	
	Always	14	4.0	
Items of psychological effect sub-dimensions	Likert	f	%	No/Yes
Item 15: I become extremely irritable	Never	100	28.6	28.6
	Rarely	106	30.3	71.4
	Sometimes	75	21.4	
	Often	40	11.4	
	Always	29	8.3	
Item 16: I feel extremely tired	Never	64	18.3	18.3
	Rarely	120	34.3	81.7
	Sometimes	86	24.6	
	Often	51	14.6	
	Always	29	8.3	
Item 17: My performance decreases	Never	75	21.4	21.4
	Rarely	161	46.0	78.6
	Sometimes	81	23.1	
	Often	24	6.9	
	Always	9	2.6	
Item 18: I get stressed	Never	71	20.3	20.3
	Rarely	140	40.0	79.7
	Sometimes	88	25.1	
	Often	31	8.9	
	Always	20	5.7	
Item 19: My desire to do sports decreases	Never	118	33.7	33.7
	Rarely	136	38.9	66.3
	Sometimes	67	19.1	
	Often	18	5.1	
	Always	11	3.1	

It was determined that the sub-dimensions of all of the weight loss methods (diet, dehydration, and ergogenic aids) and effects (physiological and psychological) were positively and moderately related to each other. Moreover, a negative and low correlation was found between the ergogenic aids and the diet sub-dimensions. This correlation shows that those using ergogenic aids do not restrict food and fluids (Table 3).

**Table 3:** The relationship between sub-dimensions of weight loss methods and effects scale among wrestlers.

Sub-dimensions of weight loss methods and effects scale		Diet	Dehydration	Ergogenic aids	Physiological effect	Psychological effect
Diet	*r*	1				
	*p*					
Dehydration	*r*	0.194^**^	1			
	*p*	0.000				
Ergogenic aids	*r*	−0.183^**^	0.223^**^	1		
	*p*	0.001	0.000			
Physiological effect	*r*	0.113^*^	0.266^**^	0.430^**^	1	
	*p*	0.034	0.000	0.000		
Psychological effect	*r*	0.210^**^	0.178^**^	0.170^**^	0.560^**^	1
	*p*	0.000	0.001	0.001	0.000	

*p < 0.05; **p < 0.01.

Of the wrestlers participating in the study, 42.86% (*n* = 150) were female and 57.14% (*n* = 200) were male. When classified according to wrestling styles, 40% (*n* = 140) were FS, 17.14% (*n* = 60) were GR and 42.86% (*n* = 150) were WW competitors. 23.43% (*n* = 82) of the wrestlers were competing in the U17, 20.00% (*n* = 70) were U20 and 56.57% (*n* = 198) were Senior category. 49.14% of the wrestlers started to lose weight one week before the competitions and wrestlers competing in the Senior category lost more weight (4.91 ± 2.18 kg) than wrestlers in the U17 (3.00 ± 1.94 kg) and U20 (3.74 ± 2.42 kg) categories (Table 4).

**Table 4:** Distribution of personal information of wrestlers.

Personal information	Categories	*f*	%	Mean ± std. deviation
Gender	Women	150	42.86	-
	Men	200	57.14	-
Wrestling styles	FS	140	40.00	-
	GR	60	17.14	-
	WW	150	42.86	-
Age Categories	U17	82	23.43	-
	U20	70	20.00	-
	Senior	198	56.57	-
How many days before the competition do you start losing weight?	<1 week	172	49.14	-
	1–2 week	77	22.00	-
	3–4 week	81	23.14	-
	≥5 week	20	5.72	-
What are your weight loss amounts according to age groups? (kg)	U17	82	-	3.00 ± 1.94
	U20	70	-	3.74 ± 2.42
	Senior	198	-	4.91 ± 2.18

When the wrestlers’ sub-dimensions of weight loss methods and effects scale were compared according to gender (Table 5) and wrestling styles (Table 6), it was determined that there was no statistically significant difference for sub-dimensions of weight loss methods and effects scale (*p* > 0.05).

**Table 5:** Comparison of sub-dimensions of weight loss methods and effects on wrestlers according to their gender.

Sub-dimensions of weight loss methods and effects scale	Gender	*N*	Mean ± std. deviation	*t*	*p*
Diet	Women	150	2.78 ± 1.00	1.607	0.109
	Men	200	2.62 ± 0.86		
Dehydration	Women	150	2.26 ± 0.62	−1.599	0.111
	Men	200	2.37 ± 0.66		
Ergogenic aids	Women	150	1.45 ± 0.74	−0.265	0.791
	Men	200	1.47 ± 0.72		
Physiological effect	Women	150	2.10 ± 0.75	1.136	0.256
	Men	200	2.01 ± 0.74		
Psychological effect	Women	150	2.40 ± 0.83	1.371	0.170
	Men	200	2.28 ± 0.79		

*p > 0.05.*

**Table 6:** Comparison of sub-dimensions of weight loss methods and effects on wrestlers according to their wrestling style.

Sub-dimensions of weight loss methods and effects scale	Wrestling style	*N*	Mean ± std. deviation	F	p
Diet	FS	140	2.66 ± 0.85	0.914	0.402
	GR	60	2.60 ± 0.88		
	WW	150	2.77 ± 1.02		
Dehydration	FS	140	2.29 ± 0.65	1.466	0.232
	GR	60	2.45 ± 0.68		
	WW	150	2.30 ± 0.62		
Ergogenic aids	FS	140	1.43 ± 0.74	0.432	0.650
	GR	60	1.53 ± 0.67		
	WW	150	1.48 ± 0.75		
Physiological effect	FS	140	2.03 ± 0.75	0.232	0.793
	GR	60	2.11 ± 0.69		
	WW	150	2.06 ± 0.76		
Psychological effect	FS	140	2.37 ± 0.86	0.208	0.812
	GR	60	2.31 ± 0.57		
	WW	150	2.32 ± 0.84		

*p > 0.05.*

When the wrestlers’ sub-dimensions of the weight loss methods and effects scale were compared according to age categories; it was determined that there was no statistically significant difference in the dehydration and ergogenic aids sub-dimensions (*p* > 0.05), whereas there was a statistically significant difference in the diet, physiological and psychological effects sub-dimensions (*p* < 0.05). According to this result, it was determined that U20 wrestlers were exposed to higher levels of dieting than U17 and Senior wrestlers. Moreover, it was determined that Senior wrestlers were exposed to lower levels of physiological and psychological effects than U17 and U20 wrestlers (Table 7).

**Table 7:** Comparison of sub-dimensions of weight loss methods and effects scale on wrestlers according to their age categories.

Sub-dimensions of weight loss methods and effects scale	Age categories	*N*	Mean ± std. deviation	*F*	*p*
Diet	U17	82	2.78 ± .94^ab^	3.576	0.029*
	U20	70	2.91 ± .92^a^		
	Senior	198	2.59 ± .92^b^		
Dehydration	U17	82	2.26 ± 0.68	1.072	0.343
	U20	70	2.41 ± 0.71		
	Senior	198	2.31 ± 0.61		
Ergogenic aids	U17	82	1.49 ± 0.71	0.089	0.914
	U20	70	1.44 ± 0.78		
	Senior	198	1.46 ± 0.73		
Physiological effect	U17	82	2.34 ± .77^a^	17.594	0.001**
	U20	70	2.27 ± .77^a^		
	Senior	198	1.86 ± .66^b^		
Psychological effect	U17	82	2.54 ± .84^a^	9.155	0.001**
	U20	70	2.55 ± .76^a^		
	Senior	198	2.18 ± .78^b^		

*p < 0.05; **p < 0.01; ^a,b^: different letter shows statistical differences between groups according to the LSD post-hoc test.

## 4 Discussion

The primary purpose of this study was to determine the weight loss methods of wrestlers before an official competition and the possible physiological and psychological effects of these methods. When the results of this study were examined, it was determined that the most preferred methods among the weight loss methods were food and fluid restriction, running with a raincoat, and using the sauna. It was determined that losing weight by spitting and using laxatives, diet pills, and diuretic pills were not preferred much (Table 1). Due to the weight loss, wrestlers reported that they experienced physiological effects such as muscle cramps (72.9%), injuries (71.1%), difficulty breathing (60.9%), increased body temperature (57.3%) and heart palpitations (48%), and psychologically, they felt extremely tired (81.7%), stressed (79.7%), decreased performance (78.6%), extremely irritability (71.4%) and decreased desire to do sports (66.3%) (Table 2).

Especially in combat sports, although the negative effects of RWL are well-known, athletes still prefer using various methods for RWL. For this reason, many scientists continue to conduct various studies on the effects of RWL. For example, when biochemical studies on RWL were examined, it was reported that RWL causes acute kidney injuries (13), failure to reduce muscle damage markers to basal levels as a metabolic response (21) and extended recovery time (22), increases in plasma osmolarity and ghrelin hormone levels (15), increases in cortisol levels, but decreases in testosterone levels (23). In addition to all these biochemical results, despite conflicting evidence, most studies show that weight loss reduces both aerobic and anaerobic performance. Aerobic performance impairments have been attributed to dehydration, decreased plasma volume, increased heart rate, hydroelectrolytic disturbances, impaired thermoregulation, and muscle glycogen depletion, whereas decreased anaerobic performance has been attributed primarily to decreased buffering capacity, glycogen depletion, and hydroelectrolytic disturbances (19). Moreover, it was reported that they were exposed to muscle cramps, injury, and heart palpitations (2) and may show hypernatremic responses due to increased plasma osmolarity (18) during RWL. When the psychological results of the studies on RWL were examined, Filaire et al. (24) reported that after a seven-day food restriction, tension, anger, fatigue, and confusion were significantly increased, and vigor was significantly decreased in judokas. Isik et al. (25) reported that as the amount of weight loss in wrestlers increased, their depression levels also increased. Slacanac et al. (26) reported that somatic anxiety increased and self-confidence, task orientation, interest/enjoy, and competence decreased due to RWL in Croatian wrestlers. The physiological and psychological results of the studies in the literature on RWL support the results of our study.

When the relationship between the weight loss methods and the sub-dimensions of the measurement tool used to determine their effects was examined in our study, it was determined that there was a positive and moderate relationship between the diet, dehydration, and ergogenic aids methods and both physiological and psychological effect sub-dimensions. Among the weight loss methods, a negative low-level relationship existed between ergogenic aids and the diet sub-dimension (Table 3). This result suggests that among the wrestlers who lost weight before a competition, those who did not restrict food and fluid preferred ergogenic aids (laxatives, diet pills and/or diuretics) to lose weight. In summary, laxative use causes motility in the bowel and diarrhea. However, long-term use can disrupt your bowel habits and cause side effects. On the other hand, diuretics increase urine volume (urine excretion rate). However, diuretics affect the nephrons and tubules in the kidneys, reduce water reabsorption and increase the volume of urine excreted. So, athletes are weighed with an empty stomach and bladder before the competition weigh-in.

The secondary purpose of this study was to determine whether the weight loss methods and their effects differed according to gender, wrestling style, and age categories. According to the results of our study, it was determined that there was no difference between the weight loss methods and the effects of these methods between both female and male wrestlers (Table 5). In addition, there was no difference between the weight loss methods and their effects in terms of wrestling styles (FS, GR, and WW) (Table 6). In a previous study, it was reported that gender was not an effective factor in achieving RWL (19). Wrestling styles were also indirectly related to gender. Because only men were divided into two as FS and GR, and only FS wrestling competitions were held for women and were called WW. However, when weight loss methods were examined according to age groups, there was a difference in the diet sub-dimension according to age categories (Table 7). According to this result, it was seen that the wrestlers in the senior category had lower diet scores and dieted at a lower level than the wrestlers in the U17 and U20 classes. When the effects of weight loss were examined, there was a difference according to age categories in both physiological and psychological effects (Table 7). It was observed that wrestlers in the senior category had lower average scores in both physiological and psychological effects sub-dimensions. This result shows that the wrestlers in the senior class experienced high levels of weight loss despite having lower levels of food and fluid restrictions (Table 4). This result suggested that they either had a longer exercise duration or were less affected physiologically and psychologically because they adapted to losing weight.

When tragic scenes related to weight loss were examined, a judoka was found dead of a heart attack in the sauna in 1996 (27). In 1997, the deaths of three college wrestlers shocked the wrestling community. Autopsies recorded the wrestlers’ cause of death as dehydration (28). At the 2000 Sydney Olympic Games, Deborah Allan’s coach cut her hair because she weighed 400 grams overweight, and even though she weighed naked, she was disqualified because she still was 100 grams overweight (29). In a very close result, Vinesh Phogat, who qualified to compete in the 50 kg final at the 2024 Paris Olympic Games, was disqualified for weighing more than 52 kg at the pre-competition weigh-in time on the final day (30). As a result, it can be considered that weight loss may detract from competition success as well as health-related physiological and psychological effects and may even cause death. Although hundreds of thousands of studies on RWL are well documented its negative effects, it is still incomprehensible that competitive athletes continue to experience RWL, which exposes them to physiological and psychological effects that disregard human health.

## 5 Conclusion

As a result of the study, it was determined that wrestlers often preferred methods such as restricting food and fluids, using a sauna, and running with a raincoat to lose weight, but they rarely preferred ergogenic aids. It was observed that they were exposed physiologically to muscle cramps, injuries, difficulty breathing, increased body temperature, and heart palpitations, and psychologically to extremely tired, stressed, decreased performance, extremely irritable, and decreased desire to do sports.

### 5.1 Suggestions

Especially in an official wrestling competition, the time between the weigh-in time and the competition is approximately 2 h, so wrestlers should avoid RWL and dehydration. If wrestlers are required to lose weight for a competition, they should perform their weight loss processes more extensively. Thus, the physiological and psychological effects of weight loss will decrease. Athletes who lose more than 5% of their body weight for a competition should consider moving up to a higher weight category to prevent them from experiencing the effects of weight loss.

### 5.2 Limitations of the study

Of course, this research also had some limitations like every study. The first limitation is that no biochemical markers were used to determine physiological and psychological effects in this study. In future studies, the data obtained through the survey should be supported by biochemical parameters. Secondly, this study used the survey technique, which was one of the quantitative research methods. In addition to quantitative research methods for these research variables in future studies, interview and/or observation analyses from qualitative research methods can be used, or mixed research methods can be used.

## Data Availability

The raw data supporting the conclusions of this article will be made available by the authors, without undue reservation.
